# Supplementary material for: Released ATP Mediates Spermatozoa Chemotaxis Promoted by Uterus-Derived Factor (UDF) in Ascaris suum
Source: Int J Mol Sci. 2022 Apr 6;23(7):4069. doi: 10.3390/ijms23074069 (PMC8999757; doi:10.3390/ijms23074069)
Supplement: Supplementary file 1 [file ijms-23-04069-s001.zip › ijms-1637514-supplementary.pdf]

Supplementary materials

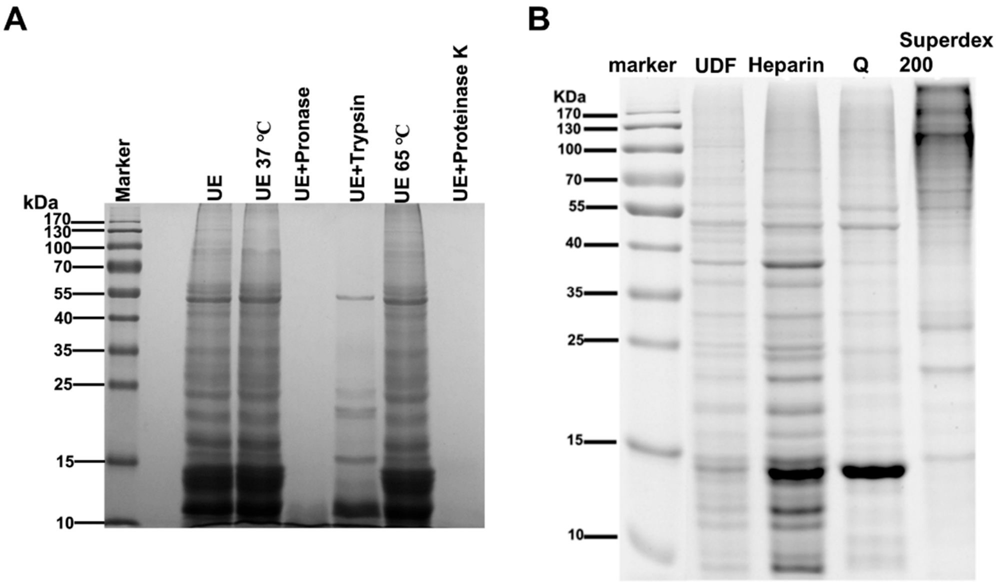

Figure S1. Characterization and purification of UDF. (A) Components in UE were degraded by proteases. Uterus extracts (UE) (5 μg) digested with Pronase, trypsin and Proteinase K were analyzed with SDS-PAGE. (B) UDF was purified with Heparin column, Q column and Superdex 200 column and active fractions in each column (5 μg protein) were detected by SDS-PAGE (12% acrylamide gel, Coomassie blue staining). The components in each column showed activity of attracting spermatozoa.

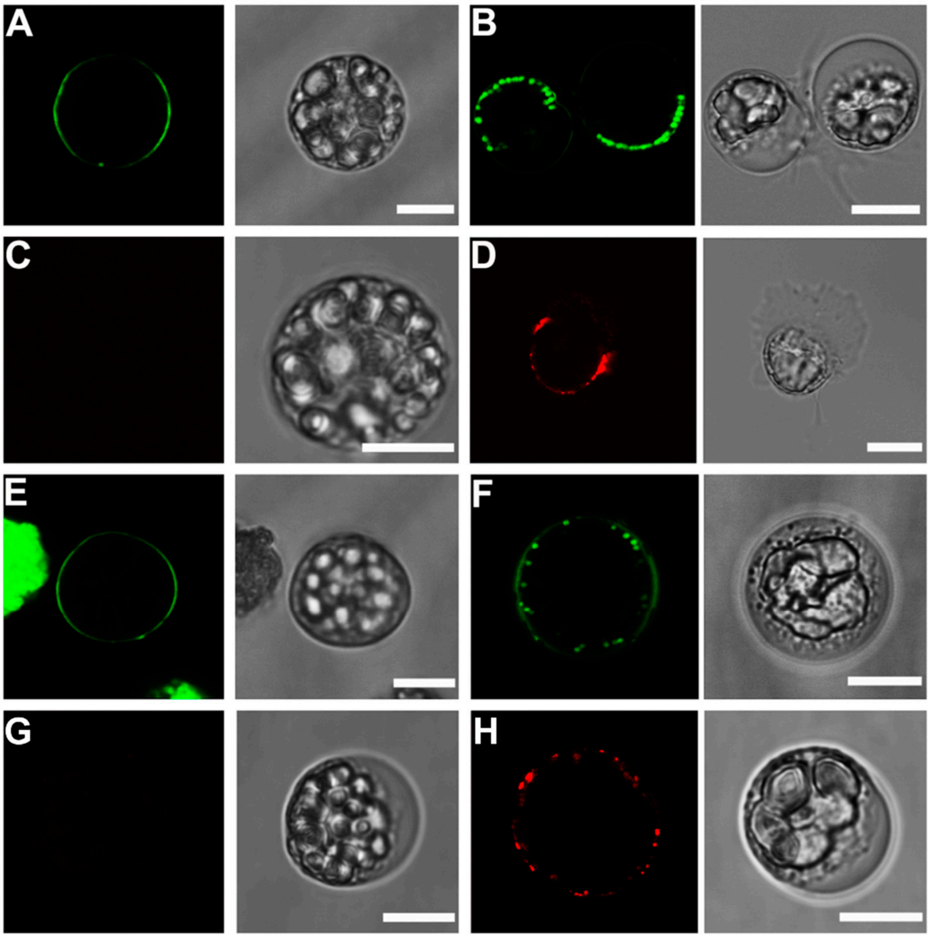

Figure S2. MO fusion plays a role in UDF binding to surface of spermatozoa. (A)(B) Spermatids (A) and spermatozoa (B) were stained with FM 1-43 (green fluorescence), a plasma membrane marker. (C)(D) Spermatids and spermatozoa were incubated with Alexa 555-UDF (red fluorescence). UDF bound spermatozoa (D) but not spermatids (C). (E)(G) Spermatids were incubated with DPC (10 μM) and activator. These sperm were stained with FM 1-43 (E) or Alexa 555-UDF (G). DPC treatment led to pseudopodia extension but not MO fusion. UDF did not bind DPC-treated sperm (G). (F)(H) Spermatids were incubated with PHE (100 μM) and activator. PHE-treated sperm were stained with FM 1-43 (F) and Alexa 555-UDF (H). PHE treatment led to MO fusion but not pseudopodia extension. UDF bound PHE-treated sperm. Scale bars represent 5 μm. The staining assays were repeated twice.

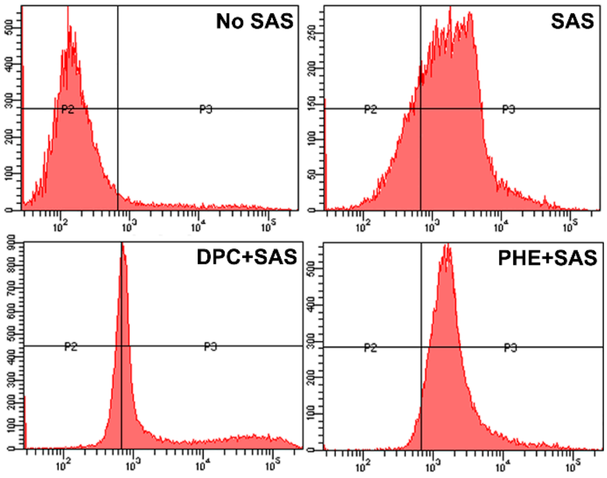

Figure S3. MO fusion is essential for UDF binding on spermatozoa. The fluorescence intensity of Alexa 555-UDF was measured by flow cytometry when sperm were treated with DPC (10 μM) which blocked MO fusion and PHE (100 μM) which did not affect MO fusion. Spermatids (no SAS) and spermatozoa (SAS) were set as control. X-axis stands for fluorescence intensity and Y-axis stands for sperm number. The results shown were based on two independent replicates.

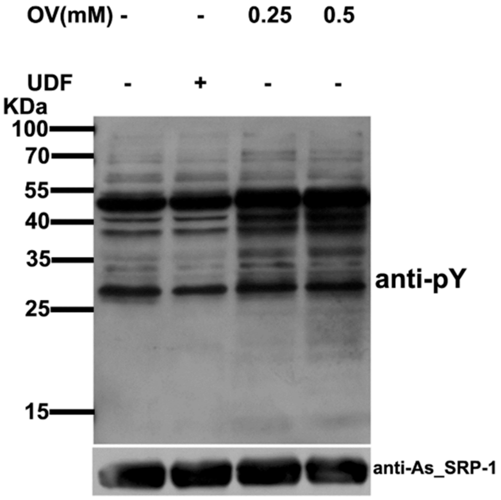

Figure S4. UDF does not affect protein tyrosine phosphorylation level. Sodium orthovanadate (OV, 0.25 mM and 0.5 mM), a tyrosine phosphatase inhibitor elevated protein tyrosine phosphorylation (PTP) level in spermatozoa. UDF (0.5 μg/μL) did not affect PTP level. The Western blotting assay was repeated for three times.
